# 786 Electrical Injury Resulting In Bilateral Upper Extremity Amputation: Improving Independence And Quality Of Life

**DOI:** 10.1093/jbcr/irac012.337

**Published:** 2022-03-23

**Authors:** Emily Adams, Brooke Murtaugh

**Affiliations:** Madonna rehabilitation hospital, Lincoln, Nebraska; Madonna Rehabilitation Hospitals, Lincoln, Nebraska

## Abstract

**Introduction:**

Fifty seven year old male who sustained an electrical burn injury that resulted in bilateral below elbow amputation began inpatient rehabilitation program. Patient was motivated to increase independence to return home prior to prosthetic fitting and training being completed. Quality options for adaptive equipment required to increase independence were not sufficient to meet patients needs, thus innovative specialty fabrication of adaptive equipment were required to meet patient’s goals of independence.

**Methods:**

Occupational therapist to use thermoplastic material to fabricate various adaptive equipment in order to address patient's goals to increase independence with self care tasks. Occupational therapist and patient to have one time consult with hospital maintenance employee to create pant hook to use for ease of donning/doffing over hips. Patient participated in daily Occupational therapy sessions focused on trialing adaptive equipment and to provide feedback for any desired changes of equipment to maximize patient progress and success with reaching independence with self cares and return to life roles.

**Results:**

Patient met all goals to become independent with self care tasks including: toileting, dressing and showering as a result of using fabricated adaptive equipment and techniques addressed during therapy sessions. Patient able to return home independently following discharge from inpatient rehabilitation stay.

**Conclusions:**

Use of easily accessible ezeform thermoplastic material can result in fabrication of adaptive equipment to increase patient's independence. It is important to maintain a creative outlook as a clinician and to continue to collaborate with our patient's to identify barriers to success and explore options to increase patient's desired independence.

